# Genome-wide association study of coronary artery calcified atherosclerotic plaque in African Americans with type 2 diabetes

**DOI:** 10.1186/s12863-017-0572-9

**Published:** 2017-12-08

**Authors:** Jasmin Divers, Nicholette D. Palmer, Carl D. Langefeld, W. Mark Brown, Lingyi Lu, Pamela J. Hicks, S. Carrie Smith, Jianzhao Xu, James G. Terry, Thomas C. Register, Lynne E. Wagenknecht, John S. Parks, Lijun Ma, Gary C. Chan, Sarah G. Buxbaum, Adolfo Correa, Solomon Musani, James G. Wilson, Herman A. Taylor, Donald W. Bowden, John Jeffrey Carr, Barry I. Freedman

**Affiliations:** 1 Department of Biostatistical Sciences, Wake Forest School of Medicine, Medical Center Boulevard, Winston-Salem, NC 27157-1053 USA; 2 Department of Biochemistry, Wake Forest School of Medicine, Winston-Salem, NC USA; 3 Center for Genomics and Personalized Medicine Research, Wake Forest School of Medicine, Winston-Salem, NC USA; 4 Department of Pathology, Wake Forest School of Medicine, Winston-Salem, NC USA; 5 Department of Epidemiology, Wake Forest School of Medicine, Winston-Salem, NC USA; 6 Department of Internal Medicine–Section on Molecular Medicine, Wake Forest School of Medicine, Winston-Salem, NC USA; 7 Department of Internal Medicine-Section on Nephrology, Wake Forest School of Medicine, Winston-Salem, NC USA; 8 Department of Radiology and Vanderbilt Center for Translation and Clinical Cardiovascular Research (VTRACC), Vanderbilt University School of Medicine, Nashville, TN USA; 9 School of Public Health Initiative, Jackson State University, Jackson, MS USA; 10 Department of Medicine, Jackson, MS USA; 11 Department of Physiology and Biophysics, University of Mississippi Medical Center, Jackson, MS USA; 12 Morehouse School of Medicine, Morehouse College, Atlanta, Georgia

**Keywords:** African Americans, Coronary artery calcified atherosclerotic plaque, Genome-wide association study, Cardiovascular disease, Type 2 diabetes, Genetics

## Abstract

**Background:**

Coronary artery calcified atherosclerotic plaque (CAC) predicts cardiovascular disease (CVD). Despite exposure to more severe conventional CVD risk factors, African Americans (AAs) are less likely to develop CAC, and when they do, have markedly lower levels than European Americans. Genetic factors likely contribute to the observed ethnic differences. To identify genes associated with CAC in AAs with type 2 diabetes (T2D), a genome-wide association study (GWAS) was performed using the Illumina 5 M chip in 691 African American-Diabetes Heart Study participants (AA-DHS), with replication in 205 Jackson Heart Study (JHS) participants with T2D. Genetic association tests were performed on the genotyped and 1000 Genomes-imputed markers separately for each study, and combined in a meta-analysis.

**Results:**

Single nucleotide polymorphisms (SNPs), rs11353135 (2q22.1), rs16879003 (6p22.3), rs5014012, rs58071836 and rs10244825 (all on chromosome 7), rs10918777 (9q31.2), rs13331874 (16p13.3) and rs4459623 (18q12.1) were associated with presence and/or quantity of CAC in the AA-DHS and JHS, with meta-analysis *p*-values ≤8.0 × 10^−7^. The strongest result in AA-DHS alone was rs6491315 in the 13q32.1 region (parameter estimate (SE) = −1.14 (0.20); *p*-value = 9.1 × 10^−9^). This GWAS peak replicated a previously reported AA-DHS CAC admixture signal (rs7492028, LOD score 2.8).

**Conclusions:**

Genetic association between SNPs on chromosomes 2, 6, 7, 9, 16 and 18 and CAC were detected in AAs with T2D from AA-DHS and replicated in the JHS. These data support a role for genetic variation on these chromosomes as contributors to CAC in AAs with T2D, as well as to variation in CAC between populations of African and European ancestry.

**Electronic supplementary material:**

The online version of this article (10.1186/s12863-017-0572-9) contains supplementary material, which is available to authorized users.

## Background

Atherosclerotic coronary artery disease remains a leading cause of death in Western societies. In 2011, cardiovascular disease (CVD) accounted for 31.3% of all-cause mortality in the US [1]. Non-invasive computed tomography (CT)-based measures of coronary artery calcified atherosclerotic plaque (CAC) are useful for risk stratification. Higher levels of CAC are associated with CVD risk and death and may refine existing algorithms used to predict CVD events [2, 3].

Individuals with type 2 diabetes (T2D) have an elevated burden of subclinical coronary atherosclerosis. The severity of CAC and suboptimal glycemic control are strong risk factors for CAC progression [4]. However, marked variation in levels of CAC and calcified atherosclerotic plaque (CP) in other vascular beds, including the carotid arteries and aorta, are observed between African and European ancestral groups [5–7]. Despite exposure to more severe conventional CVD risk factors, African Americans (AAs) have the same or lower CP levels than European Americans (EAs) [8, 9]. These risk factors include more frequent and more severe hypertension, higher LDL-cholesterol levels, and excess albuminuria [10–12]. Similar population ancestry-based CAC differences are observed in T2D individuals, with CAC levels generally increased relative to those lacking diabetes. Despite higher blood sugar, AAs with T2D have markedly lower CAC levels than similarly affected EAs [13–15].

In addition to differences in environmental exposure, abundant evidence supports inherited contributions to ethnic-specific T2D rates and CP susceptibility. A genetic basis for CAC differences based on ancestry is supported by results of the Multi-Ethnic Study of Atherosclerosis (MESA) and the African American-Diabetes Heart Study (AA-DHS) [13, 14]. Higher proportions of European ancestry were observed in AAs with higher CAC levels in both studies. AAs are an admixed population with approximately 80% African and 20% European ancestry [16]. Mapping by admixture linkage disequilibrium (MALD or admixture mapping) was performed in the AA-DHS [14]. Eleven genomic regions were suggestively or significantly linked with CAC in the AA-DHS MALD study; all demonstrated excess European ancestry in CAC-linked regions in support of European-derived risk. The present analysis reports results of the first genome-wide association study (GWAS) for CAC in AAs with T2D, the second GWAS for CAC in AAs to date.

## Methods

### Subjects

The present report contains GWAS results in 691 AAs with T2D from AA-DHS. Replication analyses were performed in AAs with T2D (*N* = 205) from the Jackson Heart Study (JHS). Diabetes was defined as fasting blood glucose (FBG) ≥126 mg/dL or a random glucose ≥200 mg/dL, history of physician diagnosis of diabetes, or use of insulin or an oral hypoglycemic agent.

#### African American-Diabetes Heart Study

As reported, AA-DHS consisted of AAs with T2D recruited from two Wake Forest School of Medicine (WFSM) studies: the family-based Diabetes Heart Study (DHS) and unrelated individuals in the AA-DHS. DHS is a cross-sectional study of EA and AA families with siblings concordant for T2D. AA-DHS started after DHS and enrolled unrelated AAs. AA-DHS objectives are to improve understanding of ethnic differences in CAC and CP in populations of African and European ancestry. T2D in AAs was diagnosed after the age of 30 years in the absence of diabetic ketoacidosis. Individuals who underwent prior coronary artery bypass surgery or coronary artery angioplasty and/or stent placement were not included in the analyses, because CAC scores could have been impacted by the procedures. Those with prior myocardial infarction (MI) or stroke were included. The final analysis included 691 unrelated AAs and AA sibpairs concordant for T2D obtained by selecting all AA-DHS participants and DHS participants that passed the quality control checks described in the genotyping section (below). The study was approved by the WFSM Institutional Review Board and all participants provided written informed consent.

#### Jackson Heart Study

The Jackson Heart Study (JHS) is a prospective population-based cohort study initiated in 2000 in the Jackson, Mississippi tri-county area. The primary objective of this study was to understand the determinants of the high prevalence of common complex diseases including CVD, T2D, obesity, chronic kidney disease, and stroke in AAs [17]. The subset of JHS participants with diabetes was between 35 and 84 years old.

### Vascular imaging

CAC was quantified by non-contrast, ECG gated, cardiac computed tomography (CT) in both studies using established methods [18]. In AA-DHS, from the start of the study in 1999, four generations of CT scanners all with ECG gating were utilized (CTi, QXi, 16Pro and VCT, GE Healthcare, Waukesha, WI). Image spatial resolution, reconstruction kernel and image technique were held constant. CAC was measured on a workstation and reported as the Agatston Score using a 90 Hounsfield Unit (HU) threshold (SmartScore GE Healthcare). Additional scoring parameters included a 130 HU threshold and 2 adjacent pixels used to define the maximum calcified lesion size; the program accounted for slice thickness. In JHS, calcified plaque was measured on a 16-channel CT system (16 Pro; GE Healthcare). The CAC score was reported as an Agatston score using a 130 HU threshold (TeraRecon Aquarius Workstation, Foster City, CA). Image analysis and quality control for both studies were performed at the same central reading center. The minimum lesion size in AA-DHS was 0.52 mm^2^ and the minimum lesion size in the Jackson Heart Study was 1 mm^2^ [19, 20].

The robustness of the CAC score as an imaging biomarker across generations of CT scanners has been previously documented [5, 19]. The calcium mass and traditional Agatston scores are two scales for measuring calcified plaque. The mass score is calibrated on a per pixel basis to an external calibration phantom (Image Analysis, Columbia, KY) which reduces noise related to the method of quantification. The Agatston score uses a threshold of 130 HU and calcified plaque below this cut-point even when present are grouped with the zero scores. This creates a group of false negatives by the design of the scoring system. The calcium mass uses a threshold of 90 and minimizes the probability of a false negative result (increased sensitivity) with a modest reduction in specificity. In AA-DHS, the Pearson and Spearman correlations between the mass score and Agatston scores were 0.97 and 0.95, respectively. However, the interquartile ranges were 397 for the mass score and 180 Agatston score. Therefore, the 90 HU CAC measure is better suited than 130 HU CAC to detect low CP levels in vascular beds. Thus, the 90 HU measure coupled with the MASS scoring algorithm we employed are more sensitive for detecting calcified plaques and reduce noise in the scoring methods. This approach improved power for the CAC study outcome. The 90 HU measure was only available in AA-DHS. Therefore, we opted for a discovery approach which was conducted in AA-DHS using the 90 HU CAC measure as the outcome, followed by replication with the 130 HU CAC in JHS. In addition, two meta-analyses were performed. The main meta-analysis used AA-DHS 90 HU CAC and 130 HU Jackson Heart Study CAC measures; the inverse variance meta-analysis was performed separately in the two studies using standardized coefficients. This approach controls for minor differences in test composition between studies (i.e., 90 HU vs 130 HU). For completeness, we also performed a second meta-analysis using the 130 HU CAC score in both study samples. Results of both analyses are reported in Table 3 and Additional file 1: Table S4 for 90 HU in AA-DHS vs. 130 HU in JHS and 130 HU in both studies, respectively.

### Genotyping

DNA in AA-DHS participants was extracted from peripheral blood using the PureGene system (Gentra Systems, Minneapolis, MN). The AA-DHS GWAS utilized the Illumina 5 M chip; the JHS GWAS genotyping was performed with the Affymetrix 6.0 chip. Genotypic data in both studies were imputed to 1000 Genomes (phase 1 version 3, cosmopolitan panel) [21].

### Quality control

Quality control (QC) checks were performed before conducting the GWAS. In AA-DHS, these checks led to the exclusion of twelve individuals from the analyses: 6 had call rates <90%, 2 had discordant self-reported and genetically determined sex, 1 had a heterozygosity score outside of the mean ± 4 times the standard error interval, 2 had the same sample identifiers and 1 had 100% European ancestry. Genome-wide association analyses were performed on 691 individuals that met the QC inclusion criteria. We used a classification scheme to rank SNPs and prioritize association results. This scheme was based on the estimated minor allele frequency (MAF), the Hardy-Weinberg equilibrium (HWE) *p*-value, and the call rate. All SNPs were analyzed; however, results are reported for common variants with a HWE *p*-value ≤10^−4^ and a call rate ≥ 95%. SNPs with these qualifications also served as the basis for our imputation efforts. SNPs with a MAF between 1 and 5% that met the HWE *p*-value and call rate thresholds were also included in imputation. Similar QC procedures were applied in JHS, which led to the exclusion of 2 subjects. The final analysis included 205 JHS study participants with T2D and available CAC measurements.

### Local and global admixture

Local ancestry estimation was performed using LAMP-ANC and HAPMIX [22, 23]. A linkage disequilibrium (LD) pruning algorithm was applied with an R-squared threshold of 0.8 to select a subset of SNPs among those that met the above QC criteria. Observed data at these SNPs were then combined with HapMap phase 3 genotypes obtained from Yoruban and CEPH samples; the HapMap samples were used as anchoring populations and were not included in the analysis. The estimation process was repeated twice in AA-DHS, once with LAMP-ANC and once with HAPMIX. Results were comparable; the distribution of Spearman correlation estimates ranged between 0.88 and 0.97. Local admixture estimation in JHS was performed with LAMP-ANC. The global ancestry proportion estimates were obtained by averaging the local ancestry estimates across the genome. These global estimates were used as covariates in the association models and are reported in Tables 1 and 2.

**Table 1:** Demographic characteristics of AA-DHS participants, by presence/absence of CAC

Variables	CAC 130 HU <10 (*N* = 346)			CAC 130 HU ≥10 (*N* = 345)			ALL (*N* = 691)			*P*-value
	Mean	SD	Median	Mean	SD	Median	Mean	SD	Median	
Age (years)	52.8	8.7	52.0	59.8	9.0	60.0	56.3	9.6	56.0	<0.0001
Female (%)	66.5			53.6			60.1			0.0006
African Ancestry proportion (%)	76.4	14.9	78.9	73.8	16.0	76.4	75.1	15.5	78.0	0.05
Diabetes duration (years)	8.4	6.1	7.0	12.2	9.3	10.0	10.3	8.1	8.0	<0.0001
HbA1c (%)	8.3	2.3	7.8	8.1	1.9	7.6	8.2	2.1	7.7	0.94
C-reactive protein (mg/dl)	1.0	1.5	0.5	0.9	1.3	0.4	0.9	1.4	0.5	0.27
Glucose (mg/dl)	154.7	68.8	138.0	148.9	67.1	132.0	151.7	68.0	136.0	0.19
Low density lipoprotein cholesterol (mg/dl)	108.5	37.4	106.0	107.1	36.6	103.0	107.8	36.9	105.0	0.46
High density lipoprotein cholesterol (mg/dl)	48.2	14.4	46.0	47.8	14.4	45.0	48.0	14.4	46.0	0.51
Triglycerides (mg/dl)	128.2	112.0	104.0	128.2	130.4	100.0	128.2	121.5	102.0	0.66
Body Mass Index (kg/m^2^)	35.9	8.6	34.6	34.4	8.2	32.9	35.1	8.4	33.7	0.02
CAC 90 (Hounsfield unit)	8.8	16.1	1.5	1212.0	1810.9	442.0	609.4	1413.3	44.0	NA
CAC 130 (Hounsfield unit)	0.8	1.8	0.0	445.1	646.1	190.5	222.6	507.5	9.5	NA
ACE inhibitor use (%)	46.8			51.3			49.1			0.25
Current smoker (%)	20.3			25.2			22.8			0.007
Past smoker (%)	31.7			40.2			36.0			0.001
Hypertension (%)	78.0			89.1			83.6			0.0001
Lipid-lowering medication (%)	39.5			53.4			46.4			0.0004

**Table 2:** Demographic characteristics of Jackson Heart Study participants with diabetes, by presence/absence of CAC

Variable	CAC 130 HU <10 (*N* = 68)			CAC 130 HU ≥10 (*N* = 137)			Full Sample (*N* = 205)			*P*-value
	Mean	SD	Median	Mean	SD	Median	Mean	SD	Median	
Age (years)	54.3	10.1	53.0	60.7	9.3	60.0	58.4	10.1	59.0	<0.0001
Female (%)	73.5			63.7			67.2			0.10
African Ancestry proportion (%)	79.9	6.5	80.7	78.1	8.4	79.5	78.8	7.8	80.0	0.21
Diabetes duration (years)	8.2	7.8	6.0	11.4	9.7	10.0	10.3	9.2	8.0	0.01
HbA1c (%)	7.7	2.0	7.2	7.9	1.8	7.6	7.8	1.9	7.4	0.06
C-reactive protein (mg/dl)	0.6	0.8	0.4	0.7	1.0	0.4	0.7	0.9	0.4	0.79
Glucose (mg/dl)	147.4	62.4	133.0	145.1	53.0	130.0	146.0	56.5	132.5	0.90
Low density lipoprotein cholesterol (mg/dl)	115.9	33.1	116.0	122.2	39.5	120.0	119.9	37.4	117.0	0.12
High density lipoprotein cholesterol (mg/dl)	50.3	13.0	47.0	48.3	13.9	45.0	49.0	13.6	46.0	0.12
Triglycerides (mg/dl)	118.5	63.1	100.0	142.0	100.3	113.0	133.6	89.5	108.0	0.12
Body Mass Index (kg/m^2^)	34.2	7.0	33.6	34.0	6.5	33.0	34.1	6.7	33.1	0.94
CAC 130 HU (Hounsfield unit)	1.2	2.4	0.0	548.4	866.1	206.4	354.8	743.3	60.0	NA
ACE inhibitor use (%)	78.0			79.8			79.2			0.73
Current smoker (%)	11.2			11.2			11.2			0.99
Past smoker (%)	20.4			30.7			27.1			0.06
Hypertension (%)	82.5			86.6			85.1			0.36
Lipid-lowering medication (%)	18.6			33.5			28.4			0.01

### Imputation

Imputation was performed using IMPUTE2 with phased haplotypic data obtained from Shapeit2 [24]. The imputation effort used all SNPs that passed the QC filters. Imputation was based on 3,436,913 and 733,318 autosomal SNPs in AA-DHS and JHS, respectively. The multi-ethnic 1000 Genomes Phase I integrated variant set release (v3) was used as the reference panel [21]. Imputation was performed separately for each study. Statistical analyses were performed on imputed SNPs that had certainty score above 90%, info score above 50% and MAF greater than 1%.

### Statistical analysis

Analyses were run using Log(CAC + 1) and CAC dichotomized (presence (CAC ≥ 10) vs. absence (CAC < 10)). The value of 1 added to the observed CAC score allowed for the inclusion all subjects, even those with a CAC score of zero. This approach is justified based on the assumption that factors governing presence of CAC may differ from those influencing amount of CAC once calcification is initiated [25]. Age, gender, global African ancestry proportion, diabetes duration, hemoglobin A1c, body mass index, smoking status, and use of lipid-lowering medication were included as covariates in the model to test for association between each SNP and CAC. Analyses were run separately in each study using the same outcome definitions based on the 90 HU CAC in AA-DHS and the 130 HU score in JHS. For the continuous outcome, linear mixed models were fitted using Genome-wide Efficient Mixed Model Analysis (GEMMA) software [26]. Generalized estimating equations were implemented to test for associations with the binary outcome. All analyses adjusted for familial relationships estimated using the Relatedness Estimation in Admixed Populations (REAP) software [27]. SNPs were tested for association using the likelihood ratio test for the overall two degrees of freedom mode of inheritance model. If the overall test of association is significant, then the three a priori genetic models (dominant, additive, and recessive) were explored; the model with the best fit for each SNP was used. Correction for this maximization was applied to account for the correlation between tests and to maintain the type 1 error rate [28–30]. This approach is consistent with Fisher’s protected least significant difference multiple comparison procedure. Sample size weighted meta-analysis was performed to compare and combine results observed from each study. Penalized regression with the *L*_*1*_ norm (LASSO) was used to identify the SNP with the strongest effect size when LD caused several SNPs to display strong association with the outcome. A cross validation approach was used to determine the shrinkage parameter for each region. SNP selection was performed only in the AA-DHS subset, the larger of the two studies, to limit confounding effects. Joint tests of association between local ancestry and genotypes with CAC were also were performed. The model used for testing for association between local ancestry and CAC was similar to the one described for the genetic association tests, with local ancestry replacing the observed or imputed genotypes. If *T*_*L*_ and *T*_*G*_ denote the test statistics associated with the local ancestry and genotypic association with CAC, the joint test of association with local ancestry and genotype at each marker was calculated as ${T}_L^2+{T}_G^2$, which follows a Chi-square distribution with 2 degrees of freedom [31]. An alternative test based on the maximum of *T*_*G*_ and *T*_*L*_ was also computed, assuming these tests follow a bivariate normal distribution with a non-zero correlation. The empirical correlation was computed using the variance-covariance matrix for 2 correlated score tests [32]. Results from these tests are shown in Additional file 2: Table S3.

### Significant effects and correction for multiple testing

The analysis involved more than 13 million directly genotyped and imputed SNPs. A strict Bonferroni correction would place the significance threshold at 1.9 × 10^−9^ for a two-sided test, a highly conservative threshold. The sample sizes required to adequately power genetic association studies in AAs at this significance threshold are not feasible for a single study (or even two). These limitations are more pronounced when the focus is on atherosclerosis in the subset of this population with T2D. We prioritized meta-analysis association results that reached an adjusted *p*-value ≤8.0 × 10^−7^, with a minimum adjusted *p*-value ≤8.0 × 10^−3^ observed in each study and prior evidence of association of the gene with CAC, T2D and other factors involved in the atherosclerosis process [33, 34]. This approach combines statistical plausibility, with nominal statistical significance from both studies, and documented prior evidence that these genomic regions have been implicated in T2D and one or more processes leading to atherosclerosis. The adjusted *p*-value (or best *p*-value) is the *p*-value associated with the minimum of the three test statistics obtained with the additive, dominant and recessive mode of inheritance models.

## Results

Table 1 displays demographic and clinical data in 691 AA-DHS participants with T2D; data in JHS participants are displayed in Table 2. Although 90 HU CAC scores were analyzed in the AA-DHS GWAS, demographic data in both tables uses the 130 HU CAC score to permit comparability. AA-DHS study participants were on average two years younger than those in JHS (56.3 ± 9.6 years vs. 58.4 ± 10.1 years; *p*-value = 6.7 × 10^−3^); had slightly higher HbA1c (8.2 ± 2.1% vs. 7.8 ± 1.9%, *p*-value = 0.02); lower proportion of African ancestry (75.1 ± 15.5% vs. 78.8 ± 7.8%, *p*-value = 0.001), and lower low density lipoprotein cholesterol (LDL) levels (107.8 ± 36.9 mg/dl vs. 119.9 ± 37.4 mg/dl, *p*-value = 4.8 × 10^−5^). At the 130 HU threshold, JHS participant were more likely to have CAC ≥ 10, compared to AA-DHS participants (49.9% in AA-DHS vs. 66.8% in JHS, *p*-value = 8.6 × 10^−6^). However, when CAC was present, AA-DHS and JHS had comparable levels (445.1 ± 646.1 vs. 548.4 ± 866.1, *p*-value = 0.11). In addition, participants in AA-DHS were less likely to be on angiotensin converting enzyme (ACE) inhibitors than JHS (49.1% vs. 79.2%, *p*-value = 3.2 × 10^−35^), but more likely to be current or former smokers (58.8% vs. 38.3%, *p*-value = 7.8 × 10^−26^) and on lipid lowering medications (46.4% vs. 28.4%, *p*-value = 6.1 × 10^−17^).

Within AA-DHS, individuals with 130 HU CAC ≥10 were on average 6 years older (*p*-value <0.0001), had longer diabetes durations (12.2 ± 9.3 years vs. 8.4 ± 6.1, *p*-value = 5.3 × 10^−5^), and were more likely to be on lipid lowering medications (53.4% vs. 39.5%, *p*-value = 2.2 × 10^−4^). Similar patterns were observed in JHS; study participants with CAC ≥10 were more likely to be older (average age difference 5.4 years, *p*-value < 0.0001), have longer diabetes durations (average difference 3.2 years, *p*-value = 0.01) and more likely to receive lipid lowering medications (33.5% vs. 18.6%, *p*-value = 0.01). The 90 HU CAC measure was more sensitive than the 130 HU CAC score at the lower end of the calcified plaque (CP) distribution; 37.7% of AA-DHS had 90 HU CAC <10 vs. 51.1% with 130 HU CAC, which supports the use of 90 HU CAC measure as the outcome in the GWAS, especially when focusing on presence of CAC. The kappa statistic measuring the agreement between the 2 measures was 0.75 with the cutoff value of 10. However, the spearman rank correlation between 90 HU CAC and 130 HU CAC was over 99% when the CP level was above 10 with both thresholds.

**Fig. 1:**
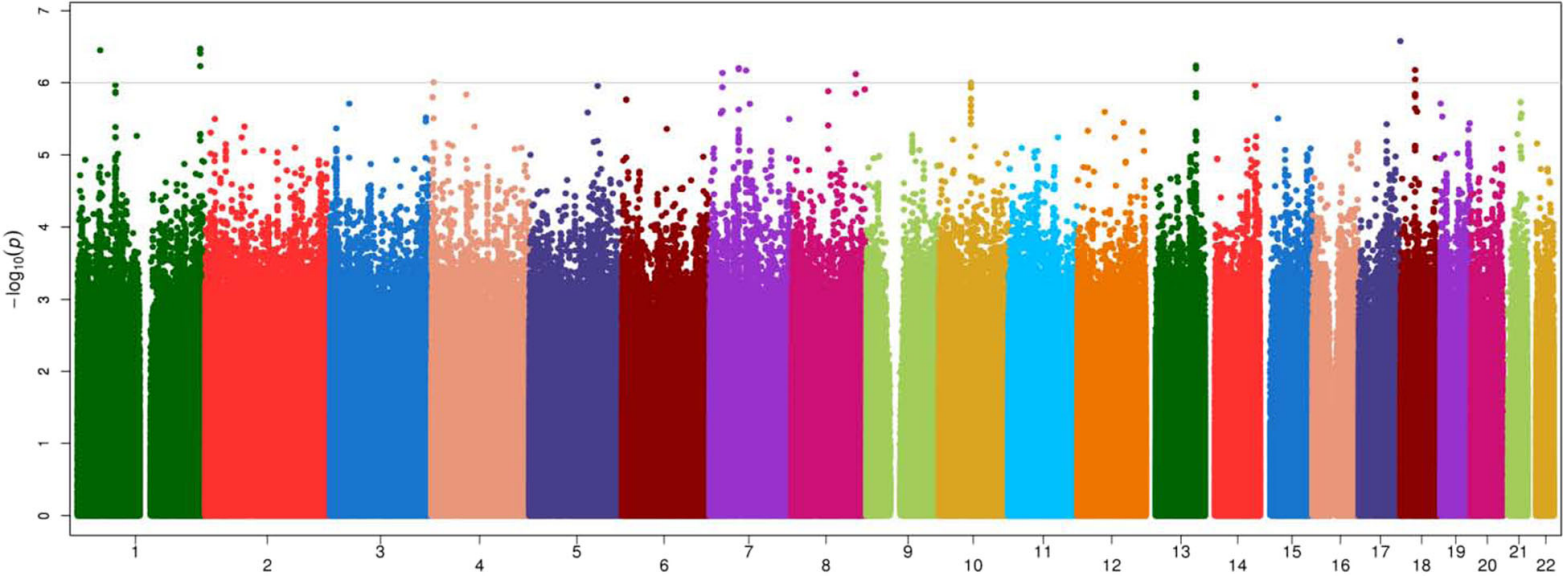
Manhattan Plot of the meta-analysis of Log(CAC + 1) between AA-DHS and JHS CAC was measured at the 90 HU threshold in AADHS and the 130 HU threshold in JHS

**Fig. 2:**
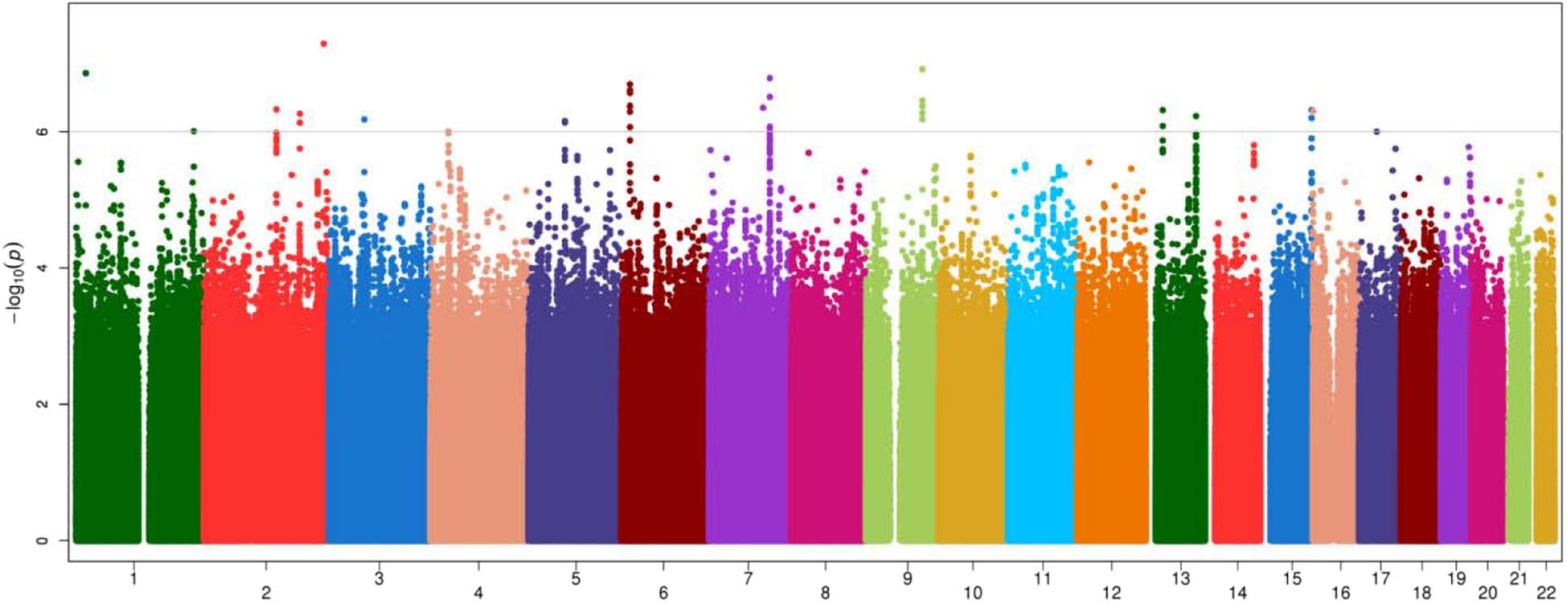
Manhattan Plot of the meta-analysis of Presence of CAC between AA-DHS and JHS CAC was measured at the 90 HU threshold in AADHS and the 130 HU threshold in JHS

**Fig. 3:**
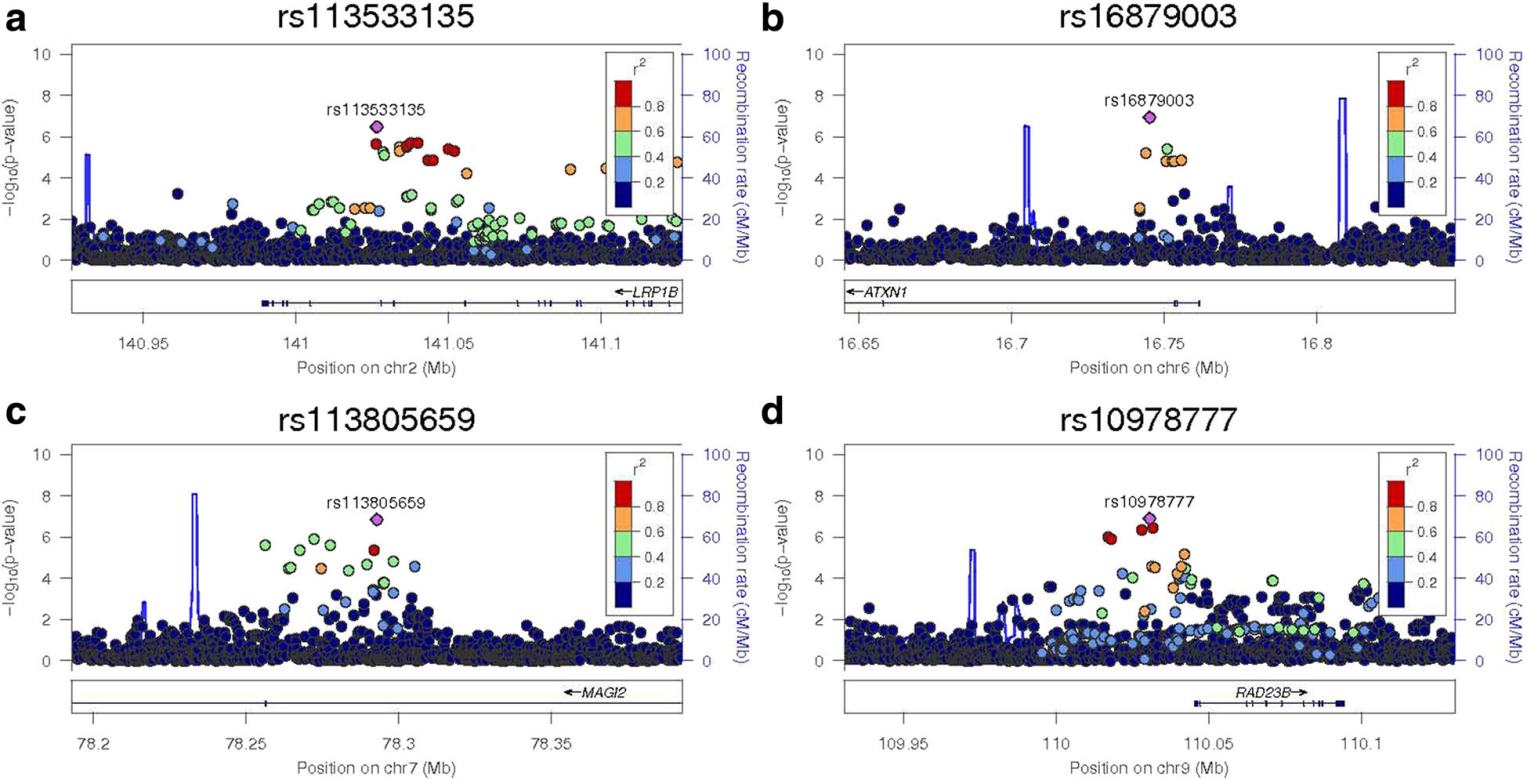
Regional association plots for the ‘sentinel’ SNP in *LRP1B, ATXN1, MAGI2,* 9q31.2 –log10 (*p*-values) are shown for all SNPs in each region with the color of circles indicating the degree of LD with the most associated SNP in the region. These regions are: **a** rs113533135 in *LRP1B*, **b** rs16879003 in in *ATXN1*, **c** rs113805659 in *MAGI2*; and **d** rs10978777 on 9q31.2. Observed *p*-values came from the meta-analysis for all SNPs

**Fig. 4:**
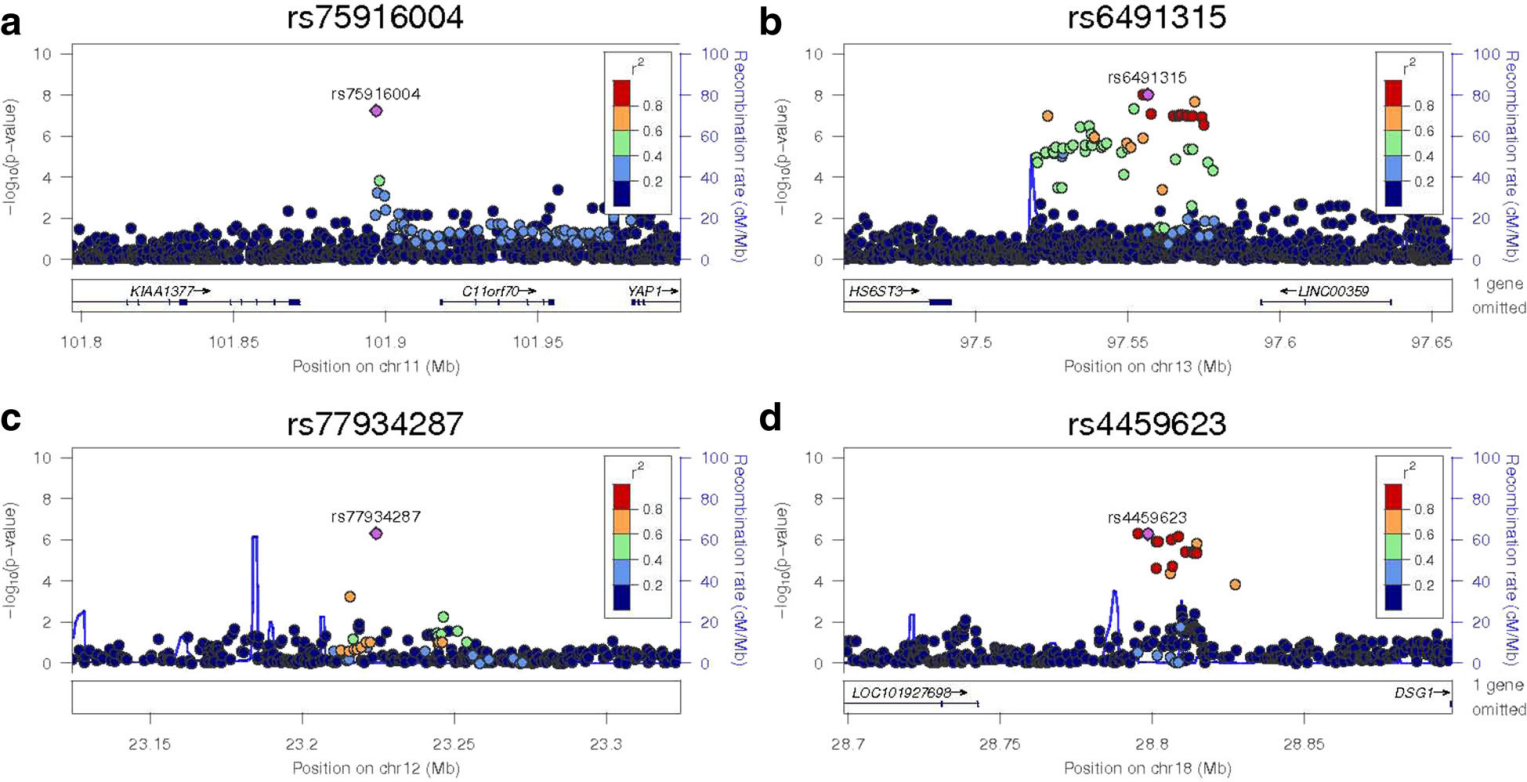
Regional association plots for the ‘sentinel’ SNP in 11q22.1, *AK094733, HS6ST3* and *DSC1.* –log10 (*p*-values) are shown for all SNPs in each region with the color of circles indicating the degree of LD with the most associated SNP in the region. These regions are: **a** rs75916004 on 11q22.1, **b** rs77934287 in chromosome 12, **c** rs6491315 in *HS6ST3* and **d** rs4459623 in *DSC1*. Observed *p*-values came from the meta-analysis for all SNPs, except rs6491315 in *HS6ST3.* This is region with the strongest result in AA-DHS; however, this signal was not replicated in JHS

Complete results of the sample size weighted meta-analysis GWAS for CAC are displayed in Figs. 1 and 2 for the continuous and binary analyses, respectively. SNPs that reach a meta-analysis *p*-value less than 10^−4^ are shown in Additional file 3: Table S1. Table 3 displays the top meta-analysis GWAS results for 90 HU CAC in AA-DHS and 130 HU CAC in JHS, limited to those with meta-analysis *p*-values ≤8.0 × 10^−7^. SNPs that were associated with both presence and amount of CAC tended to have stronger association effects with the amount of CAC modeled as Log(CAC + 1). In this case, only the association with the continuous outcome is reported. Association effects shown with presence of CAC were only observed with this outcome. We focus on six genomic regions that meet our prioritization rule. Top results for the meta-analysis of the 130 HU CAC in both AA-DHS and JHS are shown in Additional file 1: Table S4. We note that rs113805659 was the only SNP that met the statistical significance threshold in both analyses. Regional association plots for these regions are shown in Figs. 3 and 4.

**Table 3:** Summary of SNPs with meta-analysis *p*-values ≤8.0 × 10^−7^

SNP	CHR	Position	Nearest Gene	Alleles	Outcome	MOI	AA-DHS^a^			JHS^b^			Meta-Analysis	
							Estimate	SE	*P*-value	Estimate	SE	*P*-value	DE	*P*-value
rs113533135	2	141,026,436	*LRP1B*	T/C	CAC > =10 vs. CAC < 10	Dominant	0.19	0.05	1.7 × 10^−4^	0.30	0.08	3.1 × 10^−4^	–	3.3 × 10^−7^
rs6754498	2	186,817,848		A/G	Log(CAC + 1)	Dominant	0.83	0.22	1.9 × 10^−4^	1.12	0.32	5.2 × 10^−4^	++	5.5 × 10^−7^
rs9973676	2	231,818,806	*GPR55*	A/G	CAC > =10 vs. CAC < 10	Dominant	−0.20	0.05	5.8 × 10^−5^	−0.22	0.07	3.1 × 10^−3^	–	6.4 × 10^−7^
rs6898559	5	125,892,929	*ALDH7A1*	A/G	CAC > =10 vs. CAC < 10	Additive	0.09	0.03	4.1 × 10^−4^	0.18	0.04	4.3 × 10^−5^	–	5.0 × 10^−7^
rs16879003	6	16,745,239	*ATXN1*	T/C	CAC > =10 vs. CAC < 10	Additive	−0.19	0.04	7.0 × 10^−6^	−0.20	0.07	4.7 × 10^−3^	++	1.1 × 10^−7^
rs113805659	7	78,292,791	*MAGI2*	C/G	CAC > =10 vs. CAC < 10	Additive	−0.19	0.04	5.5 × 10^−6^	−0.21	0.08	7.5 × 10^−3^	–	1.4 × 10^−7^
rs117854110	7	105,243,449		T/C	Log(CAC + 1)	Dominant	−2.74	0.66	4.2 × 10^−5^	−2.72	0.91	2.9 × 10^−3^	–	4.5 × 10^−7^
rs58071836	7	118,273,539		T/C	CAC > =10 vs. CAC < 10	Additive	−0.13	0.04	1.7 × 10^−4^	−0.21	0.06	2.2 × 10^−4^	++	2.8 × 10^−7^
rs10978777*	9	110,030,590		C/G	Log(CAC + 1)	Additive	−0.63	0.16	1.3 × 10^−4^	−0.95	0.24	8.7 × 10^−5^	–	1.2 × 10^−7^
rs75916004	11	101,896,887		A/G	CAC > =10 vs. CAC < 10	Dominant	0.22	0.04	1.2 × 10^−6^	0.21	0.09	1.5 × 10^−2^	–	5.9 × 10^−8^
rs77934287	12	23,224,253	*AK094733*	A/G	CAC > =10 vs. CAC < 10	Recessive	−0.38	0.10	3.0 × 10^−4^	−0.59	0.16	1.8 × 10^−4^	–	4.8 × 10^−7^
rs448792	13	32,677,424	*FRY*	T/C	CAC > =10 vs. CAC < 10	Dominant	0.21	0.05	2.3 × 10^−5^	0.21	0.08	8.4 × 10^−3^	++	6.1 × 10^−7^
rs77757620	15	42,953,597	*STARD9*	T/C	CAC > =10 vs. CAC < 10	Additive	0.15	0.04	5.0 × 10^−5^	0.18	0.06	2.3 × 10^−3^	++	4.3 × 10^−7^
rs13331874	16	581,733	*SOLH*	A/G	CAC > =10 vs. CAC < 10	Recessive	−0.28	0.06	1.6 × 10^−6^	−0.29	0.11	1.2 × 10^−2^	++	6.3 × 10^−8^
rs4459623	18	28,798,433	*DSC1*	C/G	CAC > =10 vs. CAC < 10	Recessive	−0.32	0.09	4.1 × 10^−4^	−0.55	0.13	4.3 × 10^−5^	++	5.3 × 10^−7^

*CHR* chromosome, *MOI* Mode of Inheritance, *DE* Direction of effect (+: positive association, −: negative association)

*Upstream to ABCB1

^a^Results observed with the 90 HU CAC

^b^Results observed with the 130 HU CAC

The strongest association result was observed in the ataxin-1 (*ATXN1)* gene located on 6p22.3. Penalized regression suggests that rs16879003 had the strongest estimated effect size under the additive mode of inheritance (MOI) with parameter estimate and (standard error) -0.19(0.04) in AA-DHS and −0.20(0.07) in JHS for a meta-analysis *p*-value of 1.1 × 10^−7^. Variants in the 6p22.3 region have been associated with MI and coronary heart disease (CHD) [35].

Association was also detected within *LRP1B*, a LDL receptor gene, located on 2q22.1, a genomic region previously implicated in CHD and heart failure [36, 37]. Under the dominant MOI, the parameter estimates for the association tests between rs113533135 and presence of CAC were 0.19(0.05) in AA-DHS and 0.30(0.08) in JHS for a meta-analysis *p*-value of 3.3 × 10^−7^.

Several SNPs in the membrane-associated guanylate kinase inverted 2 gene (*MAGI*2) were strongly associated with the binary outcome of presence (versus absence) of CAC. This gene is located on chromosome 7q21.11. Conditional analysis revealed that rs113805659 had the strongest effect; under an additive mode of inheritance model that counts the number of minor alleles, the parameter estimates (standard errors) were −0.19 (0.04) in AA-DHS and −0.21 (0.08) in JHS with *p*-values of 5.5 × 10^−6^ and 7.5 × 10^−3^, respectively. The association effect was in the same direction in both studies, leading to a meta-analysis *p*-value of 1.4 × 10^−7^. Variants in *MAGI2* have been associated with the disposition index, a measure of the relationship between insulin secretion and insulin sensitivity, cell-cell and cell-matrix adhesion in Hispanics and AAs with T2D, and aortic CP in the Framingham study [38–40].

Several SNPs located in the 7q31.31 region showed significance with presence of CAC. After conditional analyses, rs58071836 had the strongest effect under an additive MOI -0.13(0.04) in AA-DHS and −0.22(0.06) in JHS for a meta-analysis *p*-value of 1.8 × 10^−7^. This chromosomal region harbors *WNT16,* a gene implicated in vascular calcification and bone mineral density (BMD) [41–43].

Similar strong association results were observed on chromosome 9 with rs10918777 located on 9q31.2, under the additive model (−0.63 (0.16), *p*-value = 1.3 × 10^−4^ in AA-DHS and −0.95 (0.24), *p*-value = 8.7 × 10^−5^ in JHS and meta-analysis *p*-value = 1.2 × 10^−7^), and on chromosome 12 with rs77934287, under the recessive model (−0.38 (0.10), *p*-value = 3.0 × 10^−4^ in AA-DHS and −0.59 (0.16), *p*-value = 1.8 × 10^−4^ in JHS and meta-analysis *p*-value = 4.8 × 10^−7^).

Nearly 20 different SNPs were found to be associated with the presence of CAC in the 18q12.1 region. These SNPs were all significant under the recessive model in both datasets. Conditional analyses showed that the number of associated SNPs is primarily due to strong LD in the region. The sentinel SNP (rs4459623) had an effect size of (−0.32 (0.09), *p* = 4.1 × 10^−4^) in AA-DHS and (−0.55 (0.13), *p* = 4.3 × 10^−5^) in JHS, for a meta-analysis *p*-value 5.3 × 10^−7^. These SNPs are in an intergenic region 25 kb distal to the desmocollin 1 gene (DSC1) and 99 kb proximal to desmoglein 1 gene (DSG1) [44, 45].

Our previous admixture mapping effort identified several regions with strong evidence for linkage with local ancestry [14]. All of these regions showed that excess European ancestry is associated with the risk and progression of CAC. However, these regions were identified using a 90 HU threshold Agatston Score, a measure that is not available in JHS. We were able to replicate these results in the AA-DHS analysis, but not in JHS. For example, the admixture mapping signal reported on chromosome 13 near 13q32.1 (LOD = 2.8) replicated with several SNPs showing statistical significance at the 10^−8^ threshold in AA-DHS alone. In the fully-adjusted model, rs6491315 had an effect size of (−1.14 (0.2), *p* = 9.1 × 10^−9^); however, the effect was non-significant in JHS (0.11 (0.36), *p*-value = 0.75), yielding a meta-analysis *p*-value 5.8 × 10^−7^. Joint models that combine local ancestry with the observed genotypic data in AA-DHS are shown in Additional file 4: Table S2 for completeness.

## Discussion

This report contains one of the few GWAS for CAC in the understudied AA population with T2D; it includes 896 subjects from two of the largest studies in this population. Several SNPs had a meta-analysis *p*-value less than 8.0× 10^−7^, albeit none reached the strict Bonferroni corrected threshold. Two SNPs, rs75916004 located on chromosome 11 in the 11p15.4 region and rs13331874 located on 16p13.3 were close, with meta-analysis *p*-values of 5.9 × 10^−8^ and 6.3 × 10^−8^, respectively. However, their *p*-values were greater than 8 × 10^−3^ in JHS. Genome-wide significant association was detected between CAC and multiple SNPs located on chromosome 13 near the *HS6ST3* gene in AA-DHS. Several other genomic regions displayed suggestive evidence of association.

The chromosome 11 and 13 GWAS peaks overlay suggestive MALD peaks for CAC in AA-DHS participants. This result suggests that a portion of the ancestry-specific risk for development of CAC lies in this genomic region. Emerging data support a genetic basis for the markedly lower levels of CAC in AAs, relative to EAs. In support of clinical relevance, reduced rates of MI are present in AAs with and without T2D, provided equal access to healthcare as EAs [46–48]. We also note that no SNPs in the 9p21 and 6p24 regions that have been reported in previous GWAS of European ancestry populations met the statistical significance criteria defined for the genetic association tests. The admixture mapping results shown in Additional file 4: Table S2 still suggest that excess European ancestry in these regions contributes to the likelihood and severity of CAC; however, the association signals were weaker.

These results observed in AAs with T2D contrast with those in a recent GWAS meta-analysis for CAC in AAs where the prevalence of T2D varied between 10.3 and 33.2% [49]. That report identified significant heritability for CAC in AAs, but failed to detect genome-wide evidence of association. Gomez et al. identified one statistically significant admixture mapping peaks on chromosome 12 and three suggestive peaks on chromosomes 6, 9, and 15 [50]. These regions did not overlap with the admixture mapping results reported in Divers et al. [14] and those described in Table 3 below. Similar to Wojczynski et al. [49], only a fraction of their sample (~28%) had T2D, which may partly explain the discordant results.

We also note that some of the admixture results observed in AA-DHS did not replicate in JHS (See Additional file 2: Table S3), and we can only speculate on why that would be the case. Possible reasons include the smaller sample size (691 vs. 205), the greater sensitivity of the 90 HU CAC measure that was only available in AA-DHS, and that hyperglycemia and T2D likely provide a modifying environmental stimulus that increases the development of CAC more prominently in individuals who are genetically susceptible to atherosclerosis. All published reports that measured CAC and other sites of CP reveal markedly higher levels in subjects with T2D, relative to those without T2D [51, 52]. Therefore, it is probable that the diabetic milieu predisposed to coronary artery atherosclerosis in our populations, even among participants at lower risk for CAC due to their higher percentage of recent African ancestry. Absence of similar environmental triggers in the Wojczynski et al. AA GWAS and Gomez et al. admixture mapping meta-analyses with lower levels of CAC could have diminished the ability to detect genetic association [49, 50].

Among the significant associations identified, SNP rs16879003 resides in the *ATXN1* gene, a member of the spinocerebellar ataxia (SCA) family. Although this protein is ubiquitously expressed, its function remains unknown. However, recent studies have linked variants in this gene with adiposity, MI and CHD [35, 53]. Based on these observations, there is support for a potential role in which variation at this locus serves as a modifier, either indirectly, i.e. metabolic syndrome, or directly as a transcriptional repressor in the development of CAC more prominently in individuals who are genetically susceptible to atherosclerosis [54].

rs113533135 is located in the *LRP1B* gene, a member of the LDL receptor family. *LRP1B* is a multi-ligand receptor that binds urokinase plasminogen activator and plasminogen activator inhibitor-1, suggesting a role in fibrinolysis and extracellular matrix remodeling [55]. The *LRP1B* gene has also been associated with insulin resistance and tumor suppression in humans and LDL cholesterol levels in rats [56–58].

rs10978777 is located in the 9q31 region upstream of the *ABCA1* gene. This gene encodes a transmembrane protein that is expressed in most cells in the body and is critical for cellular cholesterol efflux and HDL particle formation [59]. Mutations in *ABCA1* cause Tangier disease, a rare genetic disorder that is characterized by plasma HDL cholesterol concentrations <5% of normal, ~50% reduction in plasma LDL cholesterol concentrations, cholesterol accumulation in macrophages, peripheral neuropathy, and hepatosplenomegaly. Some Tangier disease kindreds have premature CHD, likely due to very low plasma HDL cholesterol levels, but some are spared, presumably due to significant reductions in plasma LDL cholesterol.

rs77934287 is located on 12p12.1, a genomic region that contains the *LOX-1* gene and *ABCC9*, a gene that has been associated with the Cantu syndrome, cardiac conduction disturbances and possibly cardiomyopathy [60, 61]. *LOX-1* encodes the lectin-like oxidized LDL receptor, which is expressed on the surface of macrophages, smooth muscle cells, and endothelial cells. The LOX-1 receptor binds and internalizes proinflammatory oxidized LDL, an initiator of macrophage lipid accumulation and endothelial cell dysfunction. *LOX-1* expression has been documented in human atherosclerotic plaques and its expression is increased by proinflammatory cytokines. *ABCC9* is a member of the superfamily of ATP-binding cassette (ABC) transporters. The ABCC9 protein is thought to form ATP-sensitive potassium channels in cardiac, skeletal, and vascular and non-vascular smooth muscle.

Multiple SNPs in the 18q12.1 region were associated with CAC in this study (Table 3). These SNPs are in an intergenic region between *DSC1* and *DSG1*, both of which belong to the cadherin superfamily of cell-cell adhesion genes that form a cluster on chromosome 18. DSC1 and DSG1 are calcium-dependent transmembrane glycoproteins involved in desmosome formation between cells. Atherogenic lipoproteins, such as oxidized LDL, that initiate atherogenesis and endothelial cell inflammation reportedly downregulate desmocollin and desmoglein expression. This may lead to endothelial barrier dysfunction and increased influx of atherogenic lipoproteins into the arterial intima of atherosclerotic lesions [62].

This study has limitations, including the small sample size and limited generalizability of results observed to AAs with longstanding T2D. Regarding the concern over sample size, it is important to note that the replication provides the ultimate protection against type 1 errors. Unfortunately, CAC in AA populations has not been studied as intensively as in EAs. When presence of T2D is considered as an additional inclusion criterion, the present study is the largest GWAS of which we are aware in AAs with T2D, and it is unlikely that many genome-wide association datasets will soon become available in this minority population.

The 90 HU CAC variable was used instead of the 130 HU CAC in the AA-DHS because of its improved sensitivity for detection calcified plaques. A sample size weighted meta-analysis was then performed to combine the observed results with JHS. An inverse variance weighted meta-analysis combining results observed with 130 HU CAC measure that is available in both studies as a sensitivity analysis. Results from these analyses are presented in Additional file 4. Overall, they show similar patterns as the main analyses, although the meta-analysis effect sizes are usually stronger when the 90 HU CAC variable is used in AA-DHS.

## Conclusion

This report contains the first GWAS results for CAC in the understudied African American population with T2D. Potential roles for *LRB1*, *ATXN1*, *MAGI2, DSC1* and *DSG1* were reported and additional support for genomic regions identified by admixture mapping was identified. Hyperglycemia increases CAC, both in prevalence and severity, and may provide a necessary environmental trigger to detect the genetic basis of coronary atherosclerosis in populations with recent African ancestry, who seem to be biologically protected from developing CP. Future functional studies of the roles of variation in these genes in human tissues/cells and transgenic animals will contribute to our understanding of genetic susceptibility to coronary atherosclerosis.

## Additional files

Additional file 1: Table S4. Meta-analysis of 130 HU CAC scores in AA-DHS and JHS. (XLSX 230 kb)

Additional file 2: Table S3. Replication of AA-DHS admixture mapping results in JHS. (XLSX 11 kb)

Additional file 3: Table S1. List of SNPs with meta-analysis *p*-value less than 10–4. (XLSX 190 kb)

Additional file 4: Table S2. Joint test of association with local ancestry and genotype. (XLSX 389 kb)

## Abbreviations

AA: African Americans
AA-DHS: African American-Diabetes Heart Study
CAC: coronary artery calcified atherosclerotic plaque
CP: Calcified Plaque
CVD: cardiovascular disease
GEMMA: Genome-wide Efficient Mixed Model Analysis
GWAS: genome-wide association study
HDL: high density lipoprotein
HU: Hounsfield Unit
JHS: Jackson Heart Study
LD: linkage disequilibrium
LDL: low density lipoprotein
MOI: mode of inheritance
REAP: Relatedness Estimation in Admixed Populations
SNP: Single nucleotide polymorphism
T2D: type 2 diabetes
